# Malingering Detection of Cognitive Impairment With the b Test Is Boosted Using Machine Learning

**DOI:** 10.3389/fpsyg.2019.01650

**Published:** 2019-07-23

**Authors:** Giorgia Pace, Graziella Orrù, Merylin Monaro, Francesca Gnoato, Roberta Vitaliani, Kyle B. Boone, Angelo Gemignani, Giuseppe Sartori

**Affiliations:** ^1^Department of Psychology, University of Padova, Padova, Italy; ^2^Department of Surgical, Medical, Molecular and Critical Area Pathology, University of Pisa, Pisa, Italy; ^3^Department of Neurology, Ca’ Foncello Hospital, Treviso, Italy; ^4^Department of Psychiatry and Biobehavioral Sciences, UCLA School of Medicine, California School of Forensic Studies, Alliant International University, Alhambra, CA, United States

**Keywords:** b Test, malingering, cognitive performance validity, mild dementia, mild cognitive impairment, Italian population

## Abstract

**Objective:** Here we report an investigation on the accuracy of the b Test, a measure to identify malingering of cognitive symptoms, in detecting malingerers of mild cognitive impairment.

**Method:** Three groups of participants, patients with Mild Neurocognitive Disorder (*n* = 21), healthy elders (controls, *n* = 21), and healthy elders instructed to simulate mild cognitive disorder (malingerers, *n* = 21) were administered two background neuropsychological tests (MMSE, FAB) as well as the b Test.

**Results:** Malingerers performed significantly worse on all error scores as compared to patients and controls, and performed poorly than controls, but comparably to patients, on the time score. Patients performed significantly worse than controls on all scores, but both groups showed the same pattern of more omission than commission errors. By contrast, malingerers exhibited the opposite pattern with more commission errors than omission errors. Machine learning models achieve an overall accuracy higher than 90% in distinguishing patients from malingerers on the basis of b Test results alone.

**Conclusions:** Our findings suggest that b Test error scores accurately distinguish patients with Mild Neurocognitive Disorder from malingerers and may complement other validated procedures such as the Medical Symptom Validity Test.

## Introduction

Recently, an increasing number of studies have been published in order to address the phenomenon of malingering and the detection of malingered cognitive symptoms. A number of investigations (e.g., Sartori et al., 2016b; Walczyk et al., 2018) indicate that malingering typically occurs in three broad domains: psychopathology, cognitive impairment, and medical illness. In the context of cognitive dysfunctions, neuropsychologists and clinical psychologists have increasingly relied on the results of neuropsychological evaluations to inform their opinions regarding the nature, extent, and credibility of claimed cognitive impairments. Clinical and research efforts have led to increasingly sophisticated and effective methods and instruments designed to detect malingering which are typically observed in most medico-legal settings. Evidence exists suggesting that external incentive to malinger typically involves financial compensation for injuries resulting in physical impairments and/or cognitive deficits. The more extensive the cognitive dysfunction is displayed, the more monetary compensation is expected and individuals have significant motive to simulate or over-exaggerate symptoms.

Critically, most of the cognitive symptoms are easily faked even by naïve non-coached examinees in order to achieve economic compensation. For the mentioned reason, it is crucial to rely on psychometric tools in order to distinguish, on an objective basis, whether neuropsychological test scores accurately reflect cognitive dysfunctions or whether individuals attempted to simulate or over-exaggerate their difficulties (Sartori et al., 2016b, 2017). While more recent techniques rely on complex computer-based tools (e.g., Sartori et al., 2016a), paper-and-pencil tests (such as the b Test investigated here) still have great practical advantages.

Faked severe cognitive impairment can be clinically detected by comparing cognitive test results with the patient everyday abilities. Unimpaired daily living activities paired with severe impairment at cognitive tests tapping on the same functions is an indication of malingering. However, mild cognitive impairments are not usually accompanied by daily living impairments and malingerers may be difficult to detect using simple strategies consisting in comparing cognitive test results (very low) and daily activities (preserved). In order to overcome this limitation, it is possible to rely on outcomes provided by the clinical research (Coin et al., 2009; Orrù et al., 2009).

One strategy used in neuropsychological testing for detecting malingering is based on the use of simple tests. As reported by the American Academy of Clinical Neuropsychology Consensus Conference Statement on the neuropsychological assessment of effort, response bias and malingering (Heilbronner et al., 2009), these tests are typically well performed with a minimum effort by patients suffering from neurologic and psychiatric diseases, unless there is a deliberate intention to perform them poorly. Most performance validity tests (PVTs) used in compensation-seeking settings are designed to detect feigned short-term memory disorders (Boone et al., 2000; Sharland and Gfeller, 2007; Young et al., 2016). Furthermore, there are also tests for detecting simulated deficits in different cognitive areas, such as in overlearned information and processing speed.

One such test is the b Test (Boone et al., 2002). It consists of a 15-page booklet: each page contains 72 stimuli including lowercase *b’s* (target stimuli) and other symbols which are used as distractors, such as lowercase *d, q,* and *p*, and *b’*s either with diagonal or extra stems. Overall, there are 255 target stimuli in the stimulus booklet: 20 in the first page, 16 in the second, and 15 in the third. These pages are repeated five times in an increasingly smaller format. The b Test requires the examinee to circle all the *b’s* that appear on each page as quickly as possible; during the task, different types of errors may be committed: omission errors (by omitting to circle target stimuli), *d* errors (by circling *d’s*), and commission errors (by circling symbols that are not *b’s,* including *d’s*). The examiner takes note of total response time, namely of the time that the participant needs for completing the test. Total errors and mean time per page are used to calculate the Effort Index Score (or E-score), which results from the equation: (total *d* errors + total commission errors) × 10 + total omission errors + mean time per page.

The b Test assesses overlearned skills and may be applied in the medico-legal setting. Patients with cerebral dysfunction who make an effort on the test are unlikely to be misclassified as non-cooperative. Patients with memory impairment may not fail on the test and this may produce less false positives than memory-based effort test. Finally, a compromised b Test performance due to the presence of overplayed symptoms and in absence of documented learning disabilities is highly suspicious of malingering (Boone et al., 2002).

The b Test may distinguish suspected malingerers from many different clinical groups including: major depressive disorder, schizophrenia, moderate or severe head injury, stroke, learning disabilities, and healthy elderly (see Boone et al., 2002).

Vilar-Lòpez et al. (2007) analyzed the performances on the b Test of a Spanish population sample with post-concussive syndrome (PCS) involved in litigation and not involved in litigation showing good sensitivity and specificity. Moreover, in an additional study (Vilar-Lòpez et al., 2008), the same authors analyzed the performances of patients with mild traumatic brain injury. The participants were divided in three groups: the first group was not involved in any compensation-seeking processes; the second group was a compensation-seeking group not suspected of malingering; the third group included patients seeking compensation who were suspected of malingering. The outcomes of the study showed that there are statistically significant differences between the groups and the malingerer versus non-malingerer classification. Similarly, Marshall et al. (2010) have investigated the validity of the b Test in detecting feigned ADHD in a large sample (*n* = 257).

Despite the promising results, Shandera et al. (2010) conducted a study on the validity of the b Test in an adult population sample suffering from mild mental retardation. The results showed unsatisfactory results when malingerers require to be distinguished from this clinical sample.

Given this result, the diagnosticity of the b Test remains still unclear, in distinguishing between Mild Neurocognitive Disorders and malingerers. To our knowledge, only Dean et al. (2009) evaluated the specificity of b test n mild dementia with unsatisfactory results.

In order to examine the potential of the b Test in classifying genuine cognitive impairment from feigned cognitive deficits in the elderly population, the present study has investigated the b Test value in an Italian sample with Mild Neurocognitive Disorders and in healthy age-matched older individuals, instructed to feign cognitive dysfunctions. Furthermore, our aim was to evaluate whether machine learning classifiers may result in an increased classification accuracy as compared to the more traditional approach based on cut-off scores.

## Materials and Methods

### Participants

Sixty-three Italian-speaking participants were recruited (36 men and 27 women, mean age: 73.43 ± 5.97 years, range: 58–87; mean education: 7.03 ± 2.78 years, range: 3–19). The sample consisted of three groups. The first group consisted of patients with mixed neurological etiology, satisfying the criteria of the Diagnostic and Statistical Manual for Mental Disorders-5 (DSM-5) (APA, 2013) for Mild Neurocognitive Disorder (Group A; *n* = 21). These patients were diagnosed according to DSM-5 criteria for Mild Neurocognitive Disorder by an expert neurologist (RV) through anamnestic interviews, neurological examinations, and neuropsychological testing. The neuropsychological assessment included: Mini Mental State Examination (MMSE, Folstein et al., 1975; Coin et al., 2009) and Frontal Assessment Battery (FAB, Dubois et al., 2000; Appollonio et al., 2005). Group A was recruited from a neurology unit in the North East of Italy.

A second group (*n* = 21, Group B) of healthy age-matched individuals was required to complete the test without specific instructions while a third group (*n* = 21, Group C) of healthy age-matched controls was instructed to respond deceitfully to the test as if they were cognitively impaired.

Healthy controls and malingerers (Group B and Group C) were recruited from two recreational centers for elderly people.

### Mild Neurocognitive Disorders

#### Participants With Mild Neurocognitive Disorder

Twenty-one participants (14 males and 7 females) were diagnosed with Mild Neurocognitive Disorder (with a MMSE score in the range 20–26). Equivalent scores on the FAB were in the range 0 and 1, with the exception of five patients who scored 2, 3, and 4. Diagnosis of the 21 clinical participants was as follows: Parkinson’s disease (*n* = 8), parkinsonism (*n* = 1), MCI (*n* = 4), Alzheimer’s disease (*n* = 3), Lewy Body Dementia (*n* = 2), mixed dementia (*n* = 1), vascular dementia (*n* = 1), and dementia related to traumatic brain injury (*n* = 1).

### Healthy Participants and Malingerers of Cognitive Impairment

#### Healthy Participants

Forty-two healthy participants were recruited. They had no history of neurological or psychiatric illnesses or substance/alcohol abuse. MMSE corrected scores were ≥ 26 and no participant obtained an equivalent score < 2 on the FAB. All participants were randomly allocated to one of the following two groups. One group (Group B: healthy controls) was given the standard instruction for the b Test (*n* = 21, 14 males and 7 females) while the other one (Group C: malingerers) received instructions to feign the b Test (*n* = 21, 8 males and 13 females) in order to fake a cognitive disorder. Specifically, the malingerers (Group C) were instructed to carry out the MMSE and FAB as best as possible and only for the b Test received the under feigning instructions (they were asked to simulate by adopting strategies in accordance with their beliefs and the common knowledge about mild cognitive deficit).

### Experimental Procedures

The b Test was administered after MMSE and FAB to all participants. Just before the experimental task, participants assigned to the malingerers group were instructed to lie about their cognitive status. To increase the compliance, participants were given the following scenario: “*You should complete the test as it would be performed by a patient suffering from mild dementia or mild cognitive impairment. In particular, pretend that I am a member of the Commission that certifies disability; you should convince me that you qualify for disability payments.”* After the completion of the test, the malingerers group was also questioned as follows: *“Describe the strategy used and explain the reason why you have chosen it.”*

Patients and healthy controls were required to carry out all the tests with their maximum effort.

### Data Analysis

Data were analyzed using nonparametric statistical analysis. Furthermore, in order to evaluate classification accuracy of the b Test and avoid overfitting, we extensively used the leave-one-out cross validation (LOOCV) procedure (Cawley and Talbot, 2010). Overfitting is an exaggerated optimistic fitting to the data derived from testing the model on the same dataset used for developing the model itself. In order to achieve realistic estimate of classification error (malingerers vs. patients), overfitting should be avoided. Overfitting is an abnormal model fitting that is usually counter using out-of-sample accuracy estimation (hold-out method), which are used as a proxy of in-field accuracies. Such out-of-sample accuracy estimations require large samples, which are difficult to collect with clinical populations. It has been shown that n-fold cross validation is a good procedure for estimating true accuracies in small samples. A special case of n-fold cross validation is the LOOCV (Cawley and Talbot, 2010), a method of choice in clinical studies (Orrù et al., 2012). In LOOCV, the statistical model is developed using only *n* − 1 examples and tested on the remaining one exemplar. The procedure is repeated rotating systematically the left out example and the out-of-sample classification error is derived from the average error of the *n* − 1 models. For this reason, we have used LOOCV in order to derive cross validated discrimination figures between malingerers and patients.

Recently, it has been shown that psychometric testing may be augmented by using, on top of more traditional statistical methods, machine learning (ML) techniques (James et al., 2013). ML has already been used to develop high-performance classification models aimed to detect malingerers (Monaro et al., 2018a,b).

Data analysis has been performed using SPSS and Weka 3.8 (Hall et al., 2009).

## Results

### Nonparametric Statistical Analysis

Means and standard deviations for age, education, and test scores are reported in Table 1. Because not all test scores were normally distributed across groups, nonparametric group comparisons (Kruskal-Wallis ANOVAs) were computed. Groups did not significantly differ in age and years of education, but they differed in the MMSE and FAB scores, with the Group A scoring significantly worse than the Group B (healthy controls) and C (malingerers) on MMSE and FAB.

**Table 1:** Demographic characteristics and performance on b Test for each group of participants and Kruskal-Wallis ANOVAs.

	Patients (Group A) (*n* = 21)	Healthy controls (Group B) (*n* = 21)	Malingerers (Group C) (*n* = 21)	Significance level (*p*)
Age (mean ± standard deviation)	74.52 ± 6.49	73.14 ± 6.22	72.62 ± 5.25	0.478
Education (mean ± standard deviation)	6.29 ± 2.14	6.67 ± 2.26	8.14 ± 3.50	0.103
MMSE (mean ± standard deviation)	23.61 ± 1.90	28.10 ± 1.18	28.52 ± 1.25	<0.0001
FAB (mean ± standard deviation)	11.71 ± 2.14	15.90 ± 1.60	16.57 ± 1.32	<0.0001
**b Test**				
Omission errors (mean ± standard deviation)	34.28 ± 17.47 *R* = 26.50	17.95 ± 10.54 *R* = 16.36	184.90 ± 76.88 *R* = 48.07	<0.0001
Commission errors (mean ± standard deviation)	22.85 ± 49.69 *R* = 28.50	1.29 ± 2.76 *R* = 15.60	412.14 ± 320.40 *R* = 47.12	<0.0001
*d* errors (mean ± standard deviation)	13.81 ± 31.88 *R* = 27.78	1.14 ± 2.71 *R* = 16.98	81.71 ± 64.73 *R* = 46.36	<0.0001
Response time (mean ± standard deviation)	1020.85 + −517,841 *R* = 35.19	634.76 ± 236.23 *R* = 19.55	981.47 ± 437.63 *R* = 37.43	0.001
E-score	468.99 ± 840.65 *R* = 29.17	82.69 ± 69.04 *R* = 14.19	5246.29 ± 3792.5 *R* = 47.95	<0.0001

As shown, groups significantly differed on all b Test scores. Table 2 reports the results of Mann-Whitney U test analyses used in pairwise comparisons on b Test data (Bonferroni-corrected significance levels were set at 0.02). Malingerers (Group C) made more commission errors (including *d* errors) and omission errors, and obtained significantly higher E-scores than controls and patients (Group B and A, respectively). Healthy controls also significantly outperformed patients on these scores. Group comparisons on response times were also significant, with controls completing the test significantly more quickly than malingerers and patients, who did not significantly differ from each other.

**Table 2:** Mann-Whitney U comparisons among groups on b Test scores.

Feature	U test	Significance level (*p*)
**Patients vs. truth-tellers**		
Omission errors	95.000	0.002
Commission errors	75.500	0.001
d errors	97.000	0.001
Total response time	76.500	0.001
E-score	56.000	<0.0001
**Patients vs. malingerers**		
Omission errors	28.000	<0.0001
Commission errors	58.500	<0.0001
d errors	79.00	<0.0001
Total response time	207.00	0.734
E-score	52.000	<0.0001
**Truth-tellers vs. malingerers**		
Omission errors	18.500	<0.0001
Commission errors	21.000	<0.0001
d errors	28.500	<0.0001
Total response time	106.000	0.004
E-score	11.000	<0.0001

Error patterns revealed that patients and controls made more omission errors than commission errors (including *d* errors), while malingerers made more commission errors in general, followed by omission errors and *d* errors.

### Classification Accuracy Between Patients and Malingerers

In applying the b Test in a medico-legal setting, most interesting is the comparison between malingerers and patients. Given that in a medico-legal setting, the individual is malingering prone, the objective is to identify whether the examinee is a real pathological case or a malingerer. For this reason, the maximum interest is in efficiently distinguishing (in our experiment) patients from malingerers on the sole basis of the b Test results. Threshold scores that classify correctly 90% of the patients for each measure (omission errors, *d* errors, commission errors, response time, and E-score along with the AUC) resulted in a high classification accuracy (see Table 3). For example, as regards the omission errors, a cut-off >56 classified 90% of the patients (Group A) and 90.4% of the malingerers (Group C) correctly.

**Table 3:** b Test score cut-offs with associated sensitivity and specificity in order to discriminate patients from simulators.

	Cut-off	Malingerers correctly classified	Patients correctly classified	Average accuracy
Omission errors	>56	90.4%	90%	90.2
Commission errors	>44	81%	90%	85.5
E-score	>881	86%	90%	88
*d* errors	>31	62%	90%	76
Total response time (sec)	>1,498	14%	90%	52

*Cut-offs reported here are computed without cross-validation and may suffer from overfitting, while average classification accuracy with E-score is 88%, the same figures resulted with leave-one-out cross-validation drops to 66%*.

This result, however, may suffer from overfitting. As reported above, in order to evaluate the effectiveness of the b Test and avoid overfitting, we tested different machine learning models using the LOOCV procedure.

The predictors used in developing the machine learning model were the following: age, education, gender, *d* errors, commission errors, omission errors, total RT (sec), E-score. The leave-one-out cross validation (LOOCV) (e.g., Vapnik and Chapelle, 2000) technique was used. Such a technique leaves one single case out of the training sample used to develop the model. After the model is developed, its accuracy is tested (out of sample) in this hold-out subject. The process is repeated for all the cases in the sample (when comparing patients and malingers, 42-1). The error is then averaged over the 42–1 computations and this average error is an estimation of the out-of-sample error. The LOOCV is the method of choice when a small number of cases are available such as, for example, in neuroimaging studies (Orrù et al., 2012).

The nine features mentioned above were entered in different machine learning classifiers, which were trained to classify every subject as belonging to one of the two categories of interest (patients and malingerers). In particular, we selected the following classifiers as representative of different categories of classifiers: Naïve Bayes, Logistic Regression, Simple Logistic regression Support Vector Machine, and Random Forest (WEKA Manual for Version 3-7-8, Bouckaert et al., 2013). Results among different classifiers are reported in Table 4.

**Table 4:** Accuracies as measured by % correct, area under the curve (AUC) and F1 obtained by five different ML classifiers in leave-one-out cross validation.

Classifier	Accuracy in LOOCV (%)	AUC	F1
Naïve Bayes	90.47	0.89	0.90
Logistic regression	90.47	0.85	0.94
Simple logistics	92.9	0.91	0.93
Support vector machine	88.09	0.88	0.92
Random forest	90.47	0.89	0.90

*Perfect classification of exemplars in the two categories has an AUC of 1 and a F1 of 1. AUC stands for area under the curve in ROC analysis and F1. Here, the input variables are those listed in* Table 3. *Some classifiers such as Simple Logistic Regression drop out the less useful predictors*.

All the classifiers based on different assumptions and representative of different classes of classifiers yielded similar accurate results with similar figures both for false positive and false negative errors.

The results reported in Table 5 refer to the comparison between patients and simulators.

**Table 5:** Comparison between patients and malingerers, correctly identified by each classifier.

Classifier	Correct classification of patients	Correct classification of malingerers
Naïve Bayes	19/21	19/21
Logistic regression	21/21	17/21
Simple logistics	21/21	18/21
Support vector machine	21/21	16/21
Random forest	20/21	18/21

Finally, a Random Forest multiclass classifier which classifies the subjects but in three classes (patients, healthy controls, and malingerers) and not in two categories as reported in Tables 4, 5 yielded the following results: (1) overall accuracy = 79.4; (2) AUC = 0.87; (3) F1 = 0.8. Patients correctly classified as aforementioned were 17/21, healthy controls were 15/21, and malingerers were 18/21.

All ML models reported above are opaque and the underlying logic that yields the final classification is not straightforward. In order to have a more clear understanding of the classifying logic, we have run a tree model ML which selects the optimal decision rule that maximizes the classification accuracy, the J48 (Quinlan, 1993), which yielded the following optimal decision rule:

*if the omission errors are < = 78*, *then the subject is classified as a patient with an accuracy equal to 95.2%;*

and

*if the omission errors are > 78*, *then the subject is classified a simulator with accuracy equal to 86%.*

The mentioned decision principle is not the best classifier but gives an easy way to understand the rule, which results in high accuracy in classifying patients and malingerers. As originally indicated by Boone (2000), omission errors are those which are more contributing in correctly distinguishing simulators from patients and also this research indicates that optimal classification could be based on a rule which is based on the number of omissions.

In addition, a correlational analysis has been used to highlight which of the predictors maximally contributes to the correct classification of patients vs. simulators. Results were the following: omission = 0.81; commission = 0.66; E-score = 0.66; *d* errors = 0.56. Random Forest also permits to sort the importance of the predictors in contributing to the accurate classification and the importance of the predictors was similar to that resulting from the correlational analysis reported above with the maximum contribution to classification coming from omission and commission errors and E-score.

## Discussion

Although clinical and research efforts have led to increasingly sophisticated methods and have yielded promising results to detect malingering, there are still significant theoretical and practical challenges in the detection of malingering, especially in the elderly population with Mild Neurocognitive Disorders. Faked severe cognitive impairment can be detected clinically by comparing low scores at cognitive test results and unimpaired functioning derived from daily living. In fact, unimpaired daily living activities paired with severe impairment at cognitive tests tapping on the same functions are indicative of malingering. However, Mild Neurocognitive Disorders are not usually accompanied by daily living impairments and malingerers may be difficult to detect using simple strategies consisting in comparing cognitive test results (very low) and daily activities (preserved).

A number of strategies are available to identify malingerers of cognitive deficits. For example, the Medical Symptom Validity Test (MSVT) is a widely used memory test with three built-in effort measures that aim to detect feigning (Green, 2004; Dandachi-FitzGerald and Merckelbach, 2013). The MSVT has attained impressively high rates of sensitivity and specificity in experimental studies that have compared controls with malingerers instructed to feign memory problems (Merten et al., 2005).

The b Test belongs to the same class of tests, the so-called performance validity tests (e.g., Rey 15 items, Reznek, 2005), which are tests that are very easy also for the highly cognitively impaired.

In order to evaluate whether the b Test can help in identifying this type of malingerers, we administered the b Test to a group of patients with Mild Neurocognitive Disorder. This group was compared to healthy controls instructed to respond deceitfully to the test (artificially producing similar patterns of cognitive impairment) and a group of healthy controls instructed to respond truthfully to the test.

Major results show that malingerers scored more poorly than controls and patients on the b Test, on all parameters derived from the test except for the total response time. Notably, patients and controls made more omission errors than commission errors, including *d* errors, while malingerers made disproportionately more commission errors, followed by omission errors and *d* errors. These findings closely mirror the strategies for feigning as reported verbally by the malingerers. In fact, half of them indicated that they made random omission and commission errors, while approximately a quarter of them indicated that they attempted to circle all targets that were not *b*’s. Half of the malingerers also reported that they deliberately slowed their response time. Therefore, malingerers may be distinguished from the truth-tellers based on their error patterns, which is radically different from those showed by patients. By contrast, patients and healthy controls have similar pattern of responses and errors. While controls performed significantly better than patients on all error scores, both groups displayed more errors of omission than commission, while, as reported above, malingerers displayed the opposite pattern.

The data reported in our study are consistent with the original validation experiments from Boone et al. (2002), which documented that it was highly unusual for genuine patients with depression, stroke, traumatic brain injury, schizophrenia, learning disability, and advanced age to misidentify non-*b*’s as *b*’s. Data from the current study extend this observation to older patients with mild dementia.

Patients in our experiment performed the b Test much more slowly than did the Boone et al. (2002) patients (which included mostly psychiatric patients). These findings are consistent with observations that cognitive slowing is prominent in early dementia (McGuinness et al., 2010) and suggest that response times have very limited value in differentiating actual versus feigned mild dementia. On the contrary, errors on over-learned information tasks appear to be much more efficient. The nature of neurological disorders associated with mild cognitive impairment may explain the reason why indexes based on time are not particularly useful in detecting malingerers.

In our study, malingerers intentionally slow down their performance. However, reduced response speed is also a feature of most neurological conditions and this may be the reason why indexes based on timing may not be able to effectively differentiate between malingerers and patients.

In addition to standard statistical analysis, whose results have been summarized above, we have applied a more advanced analysis based on ML techniques. We also have reported classification accuracies based on K-fold Cross Validation (specifically leave-one-out cross validation, LOOCV; usually regarded as the best technique for handling such problems in small samples) in order to obtain unbiased estimates of out-of-sample accuracies. This analysis indicated that malingerers may be distinguished from patients exclusively on the b Test performance with an overall accuracy of 90% or more (maximum accuracy with the Simple Logistic classifier). Different types of machine learning models showed similar results. While some classifiers have highly complex decision rules (e.g., Random Forest) others may be more intuitive for the clinician. For example, an optimal decision tree yielded the following decision rule:

*if the omission errors are < = 78*, *then the subject is classified as a patient with an accuracy equal to 95.2%;*

and

if > 78 is a simulator with accuracy equal to 86%.

Despite the lower educational level (M = 6.7, SD = 2.2), healthy controls' mean response time (M = 634.76, SD= 236.23) and commission errors (M = 1.28, SD=2.75) were similar to those reported by Boone et al. (2002). Older controls (educational level = 15.2 years; mean response times = 10.8 min; mean commission errors = 1.0). Controls in the current study committed more omission errors than in the Boone and colleagues’ sample (mean omission errors: 18.0 versus 8.0). These findings suggest that education appears to have a minor impact on b Test performance, suggesting that b Test is relatively unaffected by the examinee education level.

A limitation of the current study involves the use of instructed malingerers (also called experimental malingerers). Instructed malingerers generally produce elevated sensitivity rates because they tend to feign more excessively than their “real-world” counterparts (Boone et al., 2005). Additionally, in the current study, malingerers were instructed to feign a disease that qualifies for disability compensation, so participants may have attempted to further over-exaggerate the cognitive impairment. Sensitivity rates in our study require, therefore, future replication in medico-legal settings using participants who are spontaneously motivated to fake rather than instructed to feign cognitive disorders.

## Data Availability

The dataset used and analyzed during the current study is available from the corresponding author upon reasonable request.

## Ethics Statement

The ethics committee for Clinical Trials of the provinces of Belluno and Treviso (Italy) approved the experimental procedure. All subjects gave written informed consent in accordance with the Declaration of Helsinki.

## Author Contributions

GS and FG conceived the experiment. GP, RV, and GS designed the experimental task. GP contributed to healthy subjects data acquisition. RV and GP contributed to healthy patients data acquisition. GO and AG contributed to data analysis. GO and KB contributed to data interpretation. GP, GS, KB, and GO drafted the manuscript. All the authors revised the manuscript critically and gave the final approval for the version to be published.

### Conflict of Interest Statement

The authors declare that the research was conducted in the absence of any commercial or financial relationships that could be construed as a potential conflict of interest.
